# Forensic aspects of psychiatric rehabilitation: integrating care for forensic patients with learning disabilities and neurodevelopmental disorders

**DOI:** 10.1192/j.eurpsy.2026.10299

**Published:** 2026-07-31

**Authors:** H. Al-Taiar

**Affiliations:** Forensic Psychiatry, Royal College of Psychiatrists, Oxford, United Kingdom

## Abstract

**Abstract:**

Forensic psychiatric rehabilitation occupies a pivotal position between risk management, therapeutic intervention, and social reintegration. Individuals within forensic services who have learning disabilities and neurodevelopmental disorders (including autism spectrum disorder, ADHD, and developmental communication disorders) represent a particularly vulnerable and complex subgroup. They are overrepresented in secure settings, frequently misunderstood in the criminal justice process, and at heightened risk of prolonged admission, institutionalisation, and recidivism if rehabilitation pathways are not appropriately adapted to their cognitive and social profiles.

This presentation will examine the forensic aspects of psychiatric rehabilitation for this population, integrating clinical, ethical, and legal perspectives. It will explore how neurodevelopmental impairments affect offence pathways, including social naïvety, suggestibility, emotional dysregulation, rigidity of thinking, misinterpretation of social cues, and vulnerability to exploitation. The session will discuss how these factors influence mens rea, risk formulation, and responsivity to treatment, while emphasising that rehabilitation must move beyond containment toward meaningful skill acquisition and autonomy.

A central focus will be the development of structured, individualised rehabilitation plans that account for cognitive limitations, communication needs, and adaptive functioning deficits. Evidence-based interventions—including adapted cognitive behavioural approaches, offence-focused work tailored to intellectual ability, behavioural analysis, life-skills training, vocational preparation, and supported community transition—will be discussed. The importance of multidisciplinary collaboration, including forensic psychiatry, psychology, occupational therapy, speech and language therapy, social care, and legal professionals, will be highlighted as essential to safe and sustainable discharge planning.

The presentation will also address medico-legal considerations, including proportionality of restriction, the balance between public protection and therapeutic optimism, human rights frameworks, and the ethical imperative to avoid diagnostic overshadowing. Through illustrative case material, practical challenges such as treatment resistance, risk misinterpretation, and pathway bottlenecks will be examined, alongside strategies to enhance engagement and reduce long-term dependency on secure care.

The session aims to equip clinicians and service planners with a nuanced understanding of how forensic rehabilitation must be adapted for patients with learning disabilities and neurodevelopmental disorders, ultimately improving recovery trajectories, reducing reoffending risk, and supporting dignified reintegration into the community.

**Disclosure of Interest:**

None Declared

